# Anisotropic Elastography for Local Passive Properties and Active Contractility of Myocardium from Dynamic Heart Imaging Sequence

**DOI:** 10.1155/IJBI/2006/45957

**Published:** 2006-12-03

**Authors:** Yi Liu, Ge Wang, L. Z. Sun

**Affiliations:** ^1^ Center for X-Ray and Optical Tomography, Department of Radiology, The University of Iowa Hospitals and Clinics, Iowa City, IA 52242, USA; ^2^ Department of Biomedical Engineering, Indiana University Purdue University Indianapolis, Indianapolis, IN 46204, USA; ^3^ Department of Civil and Environmental Engineering, University of California, Irvine, CA 92697, USA

## Abstract

Major heart diseases such as ischemia and hypertrophic myocardiopathy are accompanied with significant changes in the passive mechanical properties and active contractility of myocardium. Identification of these changes helps diagnose heart diseases, monitor therapy, and design surgery. A dynamic cardiac elastography (DCE) framework is developed to assess the anisotropic viscoelastic passive properties and active contractility of myocardial tissues, based on the chamber pressure and dynamic displacement measured with cardiac imaging techniques. A dynamic adjoint method is derived to enhance the numerical efficiency and stability of DCE. Model-based simulations are conducted using a numerical left ventricle (LV) phantom with an ischemic region. The passive material parameters of normal and ischemic tissues are identified during LV rapid/reduced filling and artery contraction, and those of active contractility are quantified during isovolumetric contraction and rapid/reduced ejection. It is found that quasistatic simplification in the previous cardiac elastography studies may yield inaccurate material parameters.


Hindawi Publishing Corporation
International Journal of Biomedical Imaging
Volume 2006, Article ID 45957, Pages 1–15
DOI 10.1155/IJBI/2006/45957

### Anisotropic Elastography for Local Passive Properties and Active Contractility of Myocardium from Dynamic Heart Imaging Sequence

**Yi Liu,[1,2] Ge Wang,[1] and L. Z. Sun[3]**

[1] *Center for X-Ray and Optical Tomography, Department of Radiology, The University of Iowa Hospitals and Clinics, Iowa City, IA 52242, USA*
[2] *Department of Biomedical Engineering, Indiana University Purdue University Indianapolis, Indianapolis, IN 46204, USA*
[3] *Department of Civil and Environmental Engineering, University of California, Irvine, CA 92697, USA*

Received 26 June 2006; Accepted 19 September 2006

Recommended for Publication by Seung Wook Lee

Major heart diseases such as ischemia and hypertrophic myocardiopathy are accompanied with significant changes in the passive mechanical properties and active contractility of myocardium. Identification of these changes helps diagnose heart diseases, monitor therapy, and design surgery. A dynamic cardiac elastography (DCE) framework is developed to assess the anisotropic viscoelastic passive properties and active contractility of myocardial tissues, based on the chamber pressure and dynamic displacement measured with cardiac imaging techniques. A dynamic adjoint method is derived to enhance the numerical efficiency and stability of DCE. Model-based simulations are conducted using a numerical left ventricle (LV) phantom with an ischemic region. The passive material parameters of normal and ischemic tissues are identified during LV rapid/reduced filling and artery contraction, and those of active contractility are quantified during isovolumetric contraction and rapid/reduced ejection. It is found that quasistatic simplification in the previous cardiac elastography studies may yield inaccurate material parameters.

Copyright © 2006 Yi Liu et al. This is an open access article distributed under the Creative Commons Attribution License, which permits unrestricted use, distribution, and reproduction in any medium, provided the original work is properly cited.

### 1. INTRODUCTION

Major heart diseases including ischemia, hypertrophic myocardiopathy, and myocardial dilatation are growingly pervasive with high morbidity and mortality at tremendous social and healthcare costs [1]. It is commonly observed that heart diseases are accompanied with impaired myocardium, which typically shows increase in the passive viscoelastic stiffness and/or decrease in active contractile ability. For example, in ischemia, there is loss of blood supply with the subsequent development of localized or diffuse fibrosis. The ischemic muscle thus becomes stiff with passive properties [2–4], and its active contractility is commonly weakened [5]. In the dilatation/hypokinesis states, the myocardial contractility is weakened [6]. Such impairments reduce the heart pumping ability at normal ventricular wall stress, and require increase in stress to maintain the need of circulation. Chronic high myocardial stress may lead to further damages, and may eventually cause complete heart failure. Thus, modality development for detecting, localizing, and evaluating the impaired myocardium should have far-reaching impacts on diagnosis of heart diseases, monitoring therapy, and design of surgery.

Cardiac biomechanics [7–14] has been developed to simulate anatomically accurate heart model undergoing dynamic deformation, taking into consideration the sheet-like myofiber architecture, finite-strain deformation, nonlinearity and passive/active behaviors of myocardium. With prescribed material properties of the myocardial tissue, cardiac biomechanics calculates myocardial motion and intramural strain/stress in response to ventricular blood pressure and bioelectric stimulus. Importance of the wall stress has been well recognized, as it influences major physiological factors including the ventricular pumping performance, the energy consumption in the myocardial tissue, the coronary blood flow, the vulnerability of regions to ischemia and infarction, and the remodeling of myocardium and risk of arrhythmias [15–18]. However, stress can only be calculated from the strain with knowledge of the material properties. Inaccurate constitutive description of myocardium will necessarily lead to inaccurate wall stress, and may mislead the clinical evaluations.

There have been *in vitro* experiments to characterize the passive elastic properties of heart muscle [19–25]. While these experiments provide important knowledge of the mechanical behaviors of myocardium, the main disadvantages are that the cut specimens are not in intact condition, and that the varieties due to the diversity of location, species, sex, age and health condition of the heart, and so forth, cannot be fully addressed. Furthermore, such tests are inappropriate in clinics. Therefore, the fundamental importance of accurate constitutive description of an individual living heart urges a cost-efficient, *in vivo* and noninvasive modality that reveals the passive viscoelastic properties and active contractility of normal and abnormal myocardium.

Elastography is a biomechanical technique that quantifies the material properties of an object based on *in vivo* measurements of deformation and force, instead of *in vitro* tests with cut specimens. A comprehensive elastography framework for heart tissues should include the following key elements: material anisotropy due to the sheet-like myofiber architecture, nonlinearity and viscosity of the passive mechanical properties, bioelectricity-stimulated active contraction stress, inhomogeneity and dynamic deformation, and so forth. The pioneering cardiac elastography studies include [26–31]. Han et al. [28] and Gotteiner et al. [31] conducted a series of cardiac elastography simulations, in which the heart muscle is modeled as a uniform (homogeneous) and isotropic nonlinear material, and the heart beating is approached as being quasistatic. The 2D simulations of Creswell et al. [29] and Moulton et al. [30] further took into consideration the myocardial anisotropy. In these works, the effects of time-dependent deformation have yet to be investigated, and no attempt has been made to characterize the active contraction capability of normal and impaired heart muscles. Therefore, the resulting myocardial material parameters may not be adequate and sufficiently accurate for clinical applications.

There have been tremendous developments in high-speed, high-resolution biomedical imaging technologies. The 3D anatomic structures of a living heart can now be accurately depicted [32–35]. The laminar orthotropic myofiber architecture can be reconstructed from diffusion-tensor MRI data [36–38]. The dynamic deformation has also become measurable, for instance, Lin and Robb [34] and Eusemann et al. [35] used a CT-based dynamic spatial reconstructor to track the endocardial and epicardial displacement, Kanai et al. [39] developed ultrasonic phased-tracking method to obtain the velocity field in the heart wall. Therefore, it is time to develop a dynamic cardiac elastography methodology that takes advantages of these information-rich measurements and gives accurate material parameters for both the passive behaviors and active contractibility of normal and impaired myocardium.

The proposed DCE is to identify the material parameters of myocardium, with which the computed time-dependent displacement matches optimally to the experimentally measured displacement, as described in Sections 2.1 and 2.2. In Section 2.3, a dynamic adjoint method is derived for cost-efficient computation of the gradients of the objective function with respect to the material parameters, and is compared to the previous direct method. The overall DCE framework is described in Section 2.4. Simulations are presented in Section 3 using a numerical left ventricle (LV) phantom containing an ischemic region, whose passive stiffness is much higher than the normal tissue and the active contractility is much weaker. An orthotropic viscoelastic model is employed for the passive behavior, according to the myofiber architecture. A simplified active contraction stress model is applied. The displacement on endocardial and epicardial LV surfaces is extracted from the forward simulation at discrete time steps, and is used as measurement for DCE reconstruction. Material parameters for the passive viscoelastic behaviors are identified with the LV passive (filling) phase, as in Section 3.5, and those for the active contraction are identified with the LV active (contraction) phase, as in Section 3.6. In Section 4, simulations are conducted to investigate the effects of measurement errors with the myofiber architecture and the displacement, respectively. To demonstrate the necessity of using dynamic deformation for cardiac elastography, reconstruction assuming quasistatic deformation is conducted, and is compared with the present results. Conclusion is given in Section 5.

### 2. DYNAMIC CARDIAC ELASTOGRAPHY FRAMEWORK

In this section, an optimization-based algorithm is derived for DCE to identify the passive properties and active contractility of heart muscle. The myocardium is assumed undergoing viscoelastic deformation, involving active contraction stress. An objective function is proposed to measure the discrepancy between *measured* and *computed* displacements in a cardiac cycle. A dynamic adjoint method is developed for analytical gradients of the objective function with respect to the to-be-identified material parameters, for the purpose of improving the numerical efficiency for elastography reconstruction. A flowchart is given of the optimization-based DCE reconstruction procedure.

### *2.1. Material model and motion equation*

A biological object $\Omega^0$ is described as undergoing viscoelastic deformation, with active contraction stress. Its boundary consists of $\Gamma^0_{\mathbf{T}}$, where surface force is applied, and $\Gamma^0_{\mathbf{u}}$ on which displacement and velocity are prescribed. The displacement at location $\mathbf{x}$ and time $t$ is $\mathbf{u}(\mathbf{x},t)$, the corresponding experimental measurement is $\mathbf{U}^m(\mathbf{x},t)$. The displacement-strain relation is assumed linear, that is, the strain is $\boldsymbol{\varepsilon} = (\nabla\mathbf{u} + (\nabla\mathbf{u})^T)/2$. We use "$\cdot$" to denote time derivatives, for example, strain rate is $\dot{\boldsymbol{\varepsilon}}$ and acceleration is $\ddot{\mathbf{u}}$. The stress $\boldsymbol{\sigma}(\mathbf{x},t)$ of myocardium is calculated with a Voigt-type viscoelastic constitutive relation:

$$\boldsymbol{\sigma} = \frac{\partial W(\boldsymbol{\varepsilon};\mathbf{p}_p)}{\partial\boldsymbol{\varepsilon}} + \mathbf{C}_v(\mathbf{p}_v) : \dot{\boldsymbol{\varepsilon}} + \boldsymbol{\sigma}_f(\mathrm{Ca}^{2+},\boldsymbol{\varepsilon};\mathbf{p}_a). \quad (1)$$

The first term describes passive elastic behavior with a strain energy $W$ that depends on material parameters $\mathbf{p}_p$. The second term is a viscous stress proportional to the strain rate $\dot{\boldsymbol{\varepsilon}}$ by a forth-order tensor $\mathbf{C}_v(\mathbf{p}_v)$, where $\mathbf{p}_v$ denotes viscosity parameters. The third term is the active contraction stress $\boldsymbol{\sigma}_f$ due to the calcium concentration $Ca^{2+}$. It is described by active parameters $\mathbf{p}_a$, and depends on the local strain $\boldsymbol{\varepsilon}$. The mechanical properties for myocardium is very complex, and there may not be an accurate yet neat constitutive model [26–31]. In general, the viscous stress depends on the whole deformation history. For a heart, however, the effects of history have been "shaken down" after many beating cycles, and can be equivalently described with the current strain $\boldsymbol{\varepsilon}$ and strain rate $\dot{\boldsymbol{\varepsilon}}$, and so forth. Thus the Voigt-type viscoelastic model (1) is considered a first-order approximation. The proposed DEC itself can adapt to more complex material model.

Assuming no body force, the viscoelastic motion equation and the corresponding boundary and initial conditions are

$$\begin{gathered} \nabla \cdot \boldsymbol{\sigma} = \rho_0 \ddot{\mathbf{u}} \quad (\mathbf{x} \in \Omega^0), \\ \mathbf{n} \cdot \boldsymbol{\sigma}(\mathbf{x}, t) = \mathbf{T}(\mathbf{x}, t) \quad (\mathbf{x} \in \Gamma^0_{\mathbf{T}}), \\ \mathbf{u}(\mathbf{x}, 0) = \mathbf{U}(\mathbf{x}, 0), \qquad \dot{\mathbf{u}}(\mathbf{x}, 0) = \mathbf{V}(\mathbf{x}, 0) \quad (\mathbf{x} \in \Omega^0) \end{gathered} \tag{2}$$

for $t \in [0, T_0]$, where $\rho_0$ is the mass density, $\mathbf{T}$ is the surface force and $\mathbf{n}$ is the unit outer normal to the boundary $\Gamma^0_{\mathbf{T}}$. For a heart, $\mathbf{T}$ is well approximated as zero on the epicardial surfaces and is $-P(\mathbf{x}, t)\mathbf{n}$ on the endocardial surfaces. Here $P(\mathbf{x}, t)$ denotes the blood pressure. It changes with time and varies in the four heart chambers. The dynamic displacement $\mathbf{u}$ and its time derivative $\dot{\mathbf{u}}$ are continuous in $\Omega^0$ and prescribed on $\Gamma^0_{\mathbf{u}}$, that is, $\mathbf{u} \in \kappa = \{\mathbf{u} \mid \mathbf{u}(\mathbf{x}, t) \in C^0(\mathbf{x}) \text{ in } \Omega_0,\ \mathbf{u}(\mathbf{x}, t) = \mathbf{U}(\mathbf{x}, t) \text{ and } \dot{\mathbf{u}}(\mathbf{x}, t) = \mathbf{V}(\mathbf{x}, t) \text{ on } \Gamma^0_{\mathbf{u}}\}$. The variational weak form of (2) can be shown as

$$0 = \int_{\Omega^0} \boldsymbol{\sigma} : \boldsymbol{\varepsilon}_{\mathbf{w}} dV + \int_{\Omega^0} \rho_0 \mathbf{w} \cdot \ddot{\mathbf{u}} dV - \int_{\Gamma^0_{\mathbf{T}}} \mathbf{T} \cdot \mathbf{w} dA, \tag{3}$$

for arbitrary virtual displacement $\mathbf{w} \in \kappa_0 = \{\mathbf{u} \mid \mathbf{u}(\mathbf{x}, t) \in C^0(\mathbf{x}) \text{ in } \Omega_0,\ \mathbf{u}(\mathbf{x}, t) = \mathbf{0} \text{ and } \dot{\mathbf{u}}(\mathbf{x}, t) = \mathbf{0} \text{ on } \Gamma^0_{\mathbf{u}}\}$, and $\boldsymbol{\varepsilon}_{\mathbf{w}} = (\nabla \mathbf{w} + (\nabla \mathbf{w})^T)/2$.

### 2.2. Objective function

The elastography reconstruction is thus to find material parameters, denoted with $\mathbf{p} = \{\mathbf{p}_p, \mathbf{p}_v, \mathbf{p}_a\}$, with which the displacement $\mathbf{u}$ calculated via (1) and (2) approaches experimental measurement $\mathbf{U}^m$ optimally. This is to minimize the objective function

$$\begin{gathered} \Phi(\mathbf{p}) = \int_0^{T_0} \left( \int_{\Omega^0} \mathbf{h} \cdot \boldsymbol{\chi}_\Omega(\mathbf{x}, t) \cdot \mathbf{h} dV + \int_{\Gamma^0_{\mathbf{T}}} \mathbf{h} \cdot \boldsymbol{\chi}_\Gamma(\mathbf{x}, t) \cdot \mathbf{h} dA \right) dt, \\ \mathbf{h} = \mathbf{u}(\mathbf{x}, t; \mathbf{p}) - \mathbf{U}^m(\mathbf{x}, t), \end{gathered} \tag{4}$$

in which the time interval $[0, T_0]$ is typically a part of a cardiac cycle. Second-order tensors $\boldsymbol{\chi}_\Omega(\mathbf{x}, t)$ and $\boldsymbol{\chi}_\Gamma(\mathbf{x}, t)$ are weight functions defined in $\Omega^0$ and on $\Gamma^0_{\mathbf{T}}$, respectively. In medical applications, displacement $\mathbf{U}^m$ is typically measured at $M$ discrete time steps $t = t_I$ $(I = 1, 2, \ldots, M)$. Therefore, $\boldsymbol{\chi}_\Omega(\mathbf{x}, t)$ and $\boldsymbol{\chi}_\Gamma(\mathbf{x}, t)$ should be interpreted as $\delta$-functions with respect to time, that is, $\boldsymbol{\chi}_\Omega(\mathbf{x}, t) = \sum_{I=1}^{M} \boldsymbol{\chi}_\Omega^{(I)}(\mathbf{x}) \delta(t - t_I)$ and $\boldsymbol{\chi}_\Gamma(\mathbf{x}, t) = \sum_{I=1}^{M} \boldsymbol{\chi}_\Gamma^{(I)}(\mathbf{x}) \delta(t - t_I)$. In fact, these $\delta$-functions lead to impulsive force on the adjoint system described below.

### 2.3. Dynamic adjoint method for the gradients

The simplest optimization methods only require user-supplied objective function $\Phi(\mathbf{p})$. The material parameters $\mathbf{p}$ are searched with trial-and-error method, finite-deference gradients [30], or approximate gradients [40]. Without user-supplied gradients, the optimization-based reconstruction procedure is very time-consuming, considering the large number of iterative steps needed for convergent results and the high computational expense for solving time-dependent equation (2) in each step. The difficulty for calculating the analytic gradients $\partial\Phi/\partial\mathbf{p}$ comes from the fact that the dependency of $\mathbf{u}$ on $\mathbf{p}$ is implicit and highly nonlinear. Here, we derive a dynamic adjoint method for efficient and accurate computation of $\partial\Phi/\partial\mathbf{p}$.

Following the standard treatment for constrained optimization, the weak form (3) is introduced into the objective function (4), and a Lagrangian is formed as

$$\begin{aligned} &L(\mathbf{p}; \mathbf{u}; \mathbf{w}) \\ &= \int_0^{T_0} \begin{pmatrix} \int_{\Omega^0} \mathbf{h} \cdot \boldsymbol{\chi}_\Omega \cdot \mathbf{h} dV + \int_{\Gamma^0_{\mathbf{T}}} \mathbf{h} \cdot \boldsymbol{\chi}_\Gamma \cdot \mathbf{h} dA + \int_{\Omega^0} \boldsymbol{\sigma} : \boldsymbol{\varepsilon}_{\mathbf{w}} dV \\ + \int_{\Omega^0} \rho_0 \mathbf{w} \cdot \ddot{\mathbf{u}} dV - \int_{\Gamma^0_{\mathbf{T}}} \mathbf{T} \cdot \mathbf{w} dA \end{pmatrix} dt, \end{aligned} \tag{5}$$

where $\mathbf{u} \in \kappa$ and $\mathbf{w} \in \kappa_0$. Noting that the weak form (3) is satisfied by $\mathbf{u}$ with any $\mathbf{w} \in \kappa_0$, it can be shown that $\Phi(\mathbf{p}) = L(\mathbf{p}; \mathbf{u}, \mathbf{w})$ and $\delta\Phi = \delta L$ for arbitrary displacement fields $\mathbf{w}$, $\delta\mathbf{w}$, and $\delta\mathbf{u}$ that belong to $\kappa_0$, where "$\delta$" represents variation. This allows suitable choice for $\mathbf{w}$, with which only simple terms are kept in $\delta L$ and $\delta\Phi$. To find the optimal choice, variational derivations are conducted. First, the variation of $L(\mathbf{p}; \mathbf{u}, \mathbf{w})$ is expanded as

$$\delta L = \int_0^{T_0} \begin{pmatrix} \int_{\Omega^0} 2\mathbf{h} \cdot \boldsymbol{\chi}_\Omega \cdot \delta\mathbf{u} dV + \int_{\Gamma^0_{\mathbf{T}}} 2\mathbf{h} \cdot \boldsymbol{\chi}_\Gamma \cdot \delta\mathbf{u} dA \\ + \int_{\Omega^0} (\mathbf{L}_{\mathbf{T}} : \delta\boldsymbol{\varepsilon} + \mathbf{C}_v : \delta\dot{\boldsymbol{\varepsilon}}) : \boldsymbol{\varepsilon}_{\mathbf{w}} dV \\ + \int_{\Omega^0} \rho_0 \mathbf{w} \cdot \delta\ddot{\mathbf{u}} dV + \int_{\Omega^0} \delta_{\mathbf{p}}\boldsymbol{\sigma} : \boldsymbol{\varepsilon}_{\mathbf{w}} dV \end{pmatrix} dt, \tag{6}$$

for which the terms related to $\delta\mathbf{w}$ have been canceled out since $\delta\mathbf{w} \in \kappa_0$ and $\mathbf{u}$ satisfies the weak form (3). The forth-order tensor $\mathbf{L}_{\mathbf{T}} = \partial^2 W/\partial\boldsymbol{\varepsilon}\partial\boldsymbol{\varepsilon} + \partial\boldsymbol{\sigma}_f/\partial\boldsymbol{\varepsilon}$ is the tangent elastic modulus. The stress variation due to the change of material parameters $\mathbf{p}$ is denoted as $\delta_{\mathbf{p}}\boldsymbol{\sigma}$. By integrating by part, the terms with $\delta\ddot{\mathbf{u}}$ and $\delta\dot{\boldsymbol{\varepsilon}}$ are simplified as

$$\int_0^{T_0}\int_{\Omega^0}\rho_0\mathbf{w}\cdot\delta\ddot{\mathbf{u}}dV\,dt$$

$$=\int_0^{T_0}\int_{\Omega^0}\rho_0\ddot{\mathbf{w}}\cdot\delta\mathbf{u}dV\,dt$$

$$+\int_{\Omega^0}\rho_0\big(\mathbf{w}(\mathbf{x},T_0)\cdot\delta\dot{\mathbf{u}}(\mathbf{x},T_0)$$

$$-\dot{\mathbf{w}}(\mathbf{x},T_0)\cdot\delta\mathbf{u}(\mathbf{x},T_0)\big)dV, \quad (7)$$

$$\int_0^{T_0}\int_{\Omega^0}(\mathbf{C}_v\!:\!\delta\dot{\boldsymbol{\varepsilon}}):\boldsymbol{\varepsilon}_{\mathbf{w}}dV\,dt$$

$$=-\int_0^{T_0}\int_{\Omega^0}\delta\boldsymbol{\varepsilon}:\mathbf{C}_v:\dot{\boldsymbol{\varepsilon}}_{\mathbf{w}}dV\,dt$$

$$+\int_{\Omega^0}\delta\boldsymbol{\varepsilon}(\mathbf{x},T_0):\mathbf{C}_v(\mathbf{x}):\boldsymbol{\varepsilon}_{\mathbf{w}}(\mathbf{x},T_0)dV,$$

noting that $\delta\mathbf{u}(\mathbf{x},0)=\mathbf{0}$, $\delta\dot{\mathbf{u}}(\mathbf{x},0)=\mathbf{0}$, and $\delta\boldsymbol{\varepsilon}(\mathbf{x},0)=\mathbf{0}$. Now, with selection $\mathbf{w}(\mathbf{x},T_0)=\dot{\mathbf{w}}(\mathbf{x},T_0)=\mathbf{0}$, which yield $\boldsymbol{\varepsilon}_{\mathbf{w}}(\mathbf{x},T_0)=\mathbf{0}$, the integrals in (7) can be further simplified, and (6) becomes

$$\delta L=\int_0^{T_0}\left(\begin{aligned}&\int_{\Omega^0}(\mathbf{L_T}:\boldsymbol{\varepsilon}_{\mathbf{w}}-\mathbf{C_v}:\dot{\boldsymbol{\varepsilon}}_{\mathbf{w}}):\delta\boldsymbol{\varepsilon}+(2\mathbf{h}\cdot\boldsymbol{\chi}_\Omega+\rho_0\ddot{\mathbf{w}})\\&\cdot\delta\mathbf{u}dV+\int_{\Gamma^0_{\mathbf{T}}}2\mathbf{h}\cdot\boldsymbol{\chi}_\Gamma\cdot\delta\mathbf{u}dA+\int_{\Omega^0}\delta_{\mathbf{p}}\boldsymbol{\sigma}:\boldsymbol{\varepsilon}_{\mathbf{w}}dV\end{aligned}\right)dt. \quad (8)$$

Compared to the variational weak form (3), it is readily shown that the following choice for $\mathbf{w}$ will eliminate the first two integrals in (8) for arbitrary $\delta\mathbf{u}\in\kappa_0$, that is, $\mathbf{w}\in\kappa_0$ and

$$\nabla\cdot(\mathbf{L_T}:\boldsymbol{\varepsilon}_{\mathbf{w}}-\mathbf{C_v}:\dot{\boldsymbol{\varepsilon}}_{\mathbf{w}})+\rho_0\ddot{\mathbf{w}}=-2\mathbf{h}\cdot\boldsymbol{\chi}_\Omega\quad(\mathbf{x}\in\Omega^0),$$

$$(\mathbf{L_T}:\boldsymbol{\varepsilon}_{\mathbf{w}}-\mathbf{C_v}:\dot{\boldsymbol{\varepsilon}}_{\mathbf{w}})\cdot\mathbf{n}=-2\mathbf{h}\cdot\boldsymbol{\chi}_\Gamma\quad\text{on }\Gamma^0_{\mathbf{T}}\quad(\mathbf{x}\in\Gamma^0_{\mathbf{T}}), \quad (9)$$

$$\mathbf{w}(\mathbf{x},T_0)=\mathbf{0},\qquad\dot{\mathbf{w}}(\mathbf{x},T_0)=\mathbf{0}\quad(\mathbf{x}\in\Omega^0).$$

Note that (9) gives *end conditions* for $\mathbf{w}$, instead of initial conditions. Thus, only the last term remains in (8), that is, $\delta\Phi=\delta L=\int_0^{T_0}\int_{\Omega^0}\delta_{\mathbf{p}}\boldsymbol{\sigma}:\boldsymbol{\varepsilon}_{\mathbf{w}}dV\,dt$, and the gradients $\partial\Phi/\partial\mathbf{p}$ are

$$\frac{\partial\Phi}{\partial\mathbf{p}_p}=\int_0^{T_0}\int_{\Omega^0}\boldsymbol{\varepsilon}_{\mathbf{w}}:\left(\frac{\partial^2W}{\partial\boldsymbol{\varepsilon}\partial\mathbf{p}_p}\right)dV\,dt,$$

$$\frac{\partial\Phi}{\partial\mathbf{p}_v}=\int_0^{T_0}\int_{\Omega^0}\boldsymbol{\varepsilon}_{\mathbf{w}}:\left(\dot{\boldsymbol{\varepsilon}}:\frac{\partial\mathbf{C}_v}{\partial\mathbf{p}_v}\right)dV\,dt, \quad (10)$$

$$\frac{\partial\Phi}{\partial\mathbf{p}_a}=\int_0^{T_0}\int_{\Omega^0}\boldsymbol{\varepsilon}_{\mathbf{w}}:\left(\frac{\partial\boldsymbol{\sigma}_f}{\partial\mathbf{p}_a}\right)dV\,dt.$$

The above variational derivations reflect the original idea of the continuum-based optimality criteria technique for topology optimization [41], where $\mathbf{w}$ defined in (9) is called the *adjoint* displacement. Recent elastography implementations of the adjoint method have been made by Tardieu and Constantinescu [42] for determination of elastic coefficients from indentation experiments, and by Oberai et al. [43] and Liu et al. [44] for static isotropic and anisotropic problems, respectively.

Alternatively, the gradients $\partial\Phi/\partial\mathbf{p}$ can be calculated with a direct method. Denoting the gradient of $\mathbf{u}$ with respect to the $i$th material parameter as $\mathbf{u}_{(i)}=\partial\mathbf{u}/\partial p_i$, and the associated strain as $\boldsymbol{\varepsilon}_{(i)}=(\nabla\mathbf{u}_{(i)}+(\nabla\mathbf{u}_{(i)})^T)/2$, it is shown that

$$\frac{\partial\Phi}{\partial p_i}=\int_0^{T_0}\int_{\Omega^0}2\mathbf{h}\cdot\boldsymbol{\chi}_\Omega\cdot\mathbf{u}_{(i)}dV\,dt, \quad (11)$$

where $\mathbf{u}_{(i)}\in\kappa_0$ and is the solution of

$$\nabla\cdot\mathbf{s}_{(i)}=\nabla\cdot\left(\mathbf{L_T}:\boldsymbol{\varepsilon}_{(i)}+\mathbf{C}_v:\dot{\boldsymbol{\varepsilon}}_{(i)}+\frac{\partial\boldsymbol{\sigma}}{\partial p_i}+\frac{\partial\mathbf{C}_v}{\partial p_i}:\dot{\boldsymbol{\varepsilon}}\right)$$

$$=\rho_0\ddot{\mathbf{u}}_{(i)}\quad(\mathbf{x}\in\Omega^0),$$

$$\mathbf{n}\cdot\mathbf{s}_{(i)}=\mathbf{0}\quad(\mathbf{x}\in\Gamma^0_T), \quad (12)$$

$$\mathbf{u}_{(i)}(\mathbf{x},0)=\mathbf{0},\qquad\dot{\mathbf{u}}_{(i)}(\mathbf{x},0)=\mathbf{0}\quad(\mathbf{x}\in\Omega^0).$$

Compared to the direct method, the most favorable advantage of the adjoint method is that it calculates the gradients $\partial\Phi/\partial\mathbf{p}$ most efficiently. With suitably chosen time integral algorithm, for instance, the standard Newmark-$\beta$ scheme employed in our simulations, solutions for $\mathbf{w}$ and $\mathbf{u}$ share the same finite-element effective stiffness matrix $K_{\text{eff}}=[\beta(\Delta t)^2K_T+\gamma\Delta tC+M]$ ($\Delta t$ is time step, $\beta=1/12$ and $\gamma=1/2$ are used) and its Cholesky factorization at any time step, which are most time consuming in finite-element computations. Note that matrix $K_T$ corresponds to the tangent elastic modulus $\mathbf{L_T}$, $C$ corresponds to viscosity tensor $\mathbf{C}_v$, and $M$ is the mass matrix. Therefore, solving (9) for $\mathbf{w}$ and then integrating (10) for $\partial\Phi/\partial\mathbf{p}$ just add a small fraction of cost to the solution for $\mathbf{u}$ (2), which is required in optimization-based elastography reconstruction. In contrast, the direct method (11) and (12) involves solving dynamic displacement $\mathbf{u}_{(i)}$ for every $\partial\Phi/\partial p_i$. When the number of material parameters increases, the computational expense with the direct gradient method increases proportionally, while that with the proposed adjoint method rises slightly only because more integrals are calculated (10). The tradeoff for using adjoint method is additional complexity in programming. Because the solution for $\mathbf{w}$ (9) starts at $T_0$ and ends at 0, it needs a *backward* numerical scheme, which must be consistent with the forward scheme for $\mathbf{u}$. The method also needs additional memory to store the factorization of $K_{\text{eff}}$ at every time step. Recently, Oberai et al. [45] gave an in-detail evaluation on the numerical efficiency of static elastography reconstructions using the adjoint method.

### 2.4. Optimization-based DCE reconstruction procedure

The DCE framework involves data acquisition, material modeling, and reconstruction of material parameters, shown

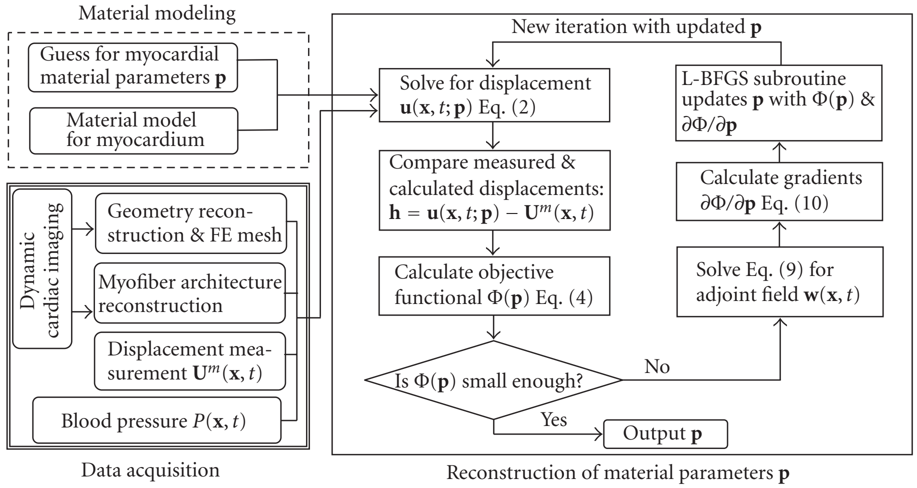

Figure 1: Flowchart for the DCE reconstruction procedure with L-BFGS optimization subroutine and user-supplied gradients.

with the flowchart in Figure 1. Data acquisition includes reconstruction of the anatomic structures of a heart and the myofiber architecture, extracting displacement data from dynamic cardiac imaging sequence, and measurement of the blood pressures and calcium kinetics. In this work, data acquisition is considered well ready (e.g., [33–38]). Formulas for the passive and active stresses are chosen, with material parameters $\mathbf{p} = \{\mathbf{p}_p, \mathbf{p}_v, \mathbf{p}_a\}$ to be reconstructed. The reconstruction procedure is optimization-based, making use of a limited-memory BFGS (L-BFGS) optimization subroutine [46], for which user-supplied gradients are required. When solving $\mathbf{u}$ with finite-element method, the factorization of $K_{\text{eff}}$ is stored at each time step, and is used for the adjoint displacement $\mathbf{w}$.

### 3. SIMULATIONS

This section presents DCE simulations with a three-dimensional left ventricle (LV) phantom containing an ischemic region, demonstrating the validity and utility of the proposed algorithm. The passive stress-strain relation is assumed linear viscoelastic, and is orthotropic according to the myofiber architecture. A simplified model is employed for the active stress. Forward computation is first carried on the phantom undergoing filling and contraction motions. Displacement on the endocardial and epicardial LV surfaces is then extracted from the forward results at discrete time steps, and is used as measurement for DCE reconstruction of the passive and active parameters of the normal and ischemic tissues.

### *3.1. LV phantom and myofiber architecture*

Our DCE simulations use a 3D LV phantom, as shown in Figures 2(a) and 2(b). To approximate the shape of a left ventricle (e.g., [47]), a thick-wall hollow ellipsoid is employed, whose surfaces are defined by

$$\left(\frac{x}{a}\right)^2 + \left(\frac{y}{b}\right)^2 + \left(\frac{z}{c}\right)^2 = \lambda^2 (a = 3.6, b = 4, c = 5), \quad (13)$$

where $\lambda = \lambda_{\text{epi}} = 1$ for the epicardial surface and $\lambda = \lambda_{\text{endo}} = 0.6875$ for the endocardial surface. The length unit is cm. The base is $z = z_{\text{Base}} = 2.5$ cm, and the apex is $z = z_{\text{Apex}} = -5.0$ cm. Shown with Figure 2(a), a red region is embedded into the wall, simulating an ischemic region. Clinically, the location, size, and shape of an impaired region can be 3D depicted from medical imaging measurements.

The myocardial tissue exhibits laminar histological structure [36–38], and is described with the local myofiber structure-based material axes $(\mathbf{n}_f, \mathbf{n}_s, \mathbf{n}_n)$ as schematically shown in Figures 2(b) and 2(c), where $\mathbf{n}_f$ is the fiber direction, $\mathbf{n}_s$ is the cross-fiber direction in the plane of myofiber layer, and $\mathbf{n}_n = \mathbf{n}_f \times \mathbf{n}_s$. These material axes vary continuously from epicardium to endocardium and from base to apex. The present simulations use a mathematical approximation to the myocardial architecture. At a point $(x, y, z)$ in the LV wall, local coordinates $(\mathbf{e}_h, \mathbf{e}_\eta, \mathbf{e}_\xi)$ are defined (Figure 2(b)) as: $\mathbf{e}_h$ is along the local thickness direction $(x/a^2, y/b^2, z/c^2)$; $\mathbf{e}_\eta$ is along direction $(-y/b^2, x/a^2, 0)$, that is, it lies in the $x - y$ plane and is perpendicular to $\mathbf{e}_h$; the third direction is $\mathbf{e}_\xi = \mathbf{e}_h \times \mathbf{e}_\eta$. In consistency with the descriptions of LeGrice et al. [36] and Costa et al. [48], and as schematically shown in Figure 2(c), the myocardial fiber is well approximated as lying in the tangential plane $(\mathbf{e}_\eta, \mathbf{e}_\xi)$, that is, $\mathbf{n}_f = \cos\alpha \mathbf{e}_\eta + \sin\alpha \mathbf{e}_\xi$, where $\alpha$ is the fiber angle. There is a sheet angle $\beta$ between $\mathbf{n}_s$ and $\mathbf{e}_h$, so that $\mathbf{n}_s = \cos\beta \mathbf{e}_h + \sin\beta(\mathbf{e}_h \times \mathbf{n}_f)$. Finally, $\mathbf{n}_n = \mathbf{n}_f \times \mathbf{n}_s$. Angles $\alpha$ and $\beta$ change transmurally and from base to apex. According to the experiment observations [36, 48], a linear transmural distribution is assumed for $\alpha$ and a quadratic distribution is

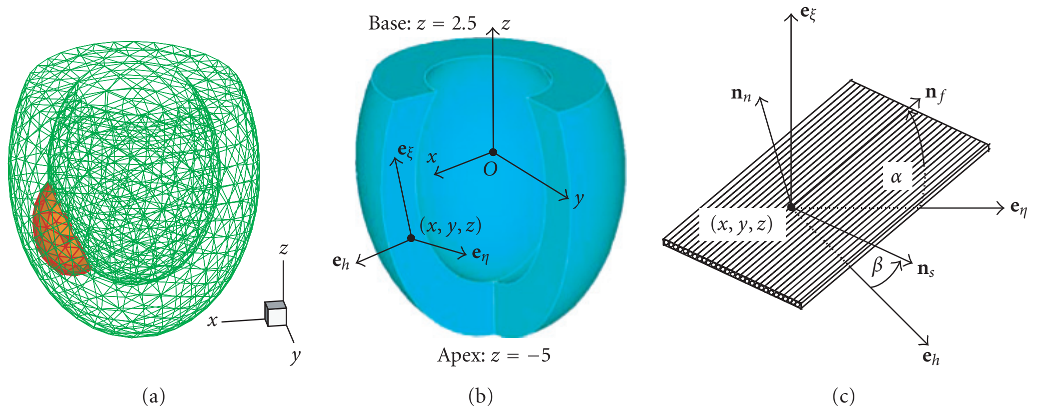

Figure 2: (a) A thick-wall hollow ellipsoidal LV phantom. Finite element mesh is shown on the surfaces. The red zone imitates an ischemic myocardial region; (b) local coordinates $(\mathbf{e}_h, \mathbf{e}_\eta, \mathbf{e}_\xi)$ at point $(x, y, z)$; (c) schematic plot of the spatial myofiber architecture described by the local material axes $(\mathbf{n}_f, \mathbf{n}_s, \mathbf{n}_n)$. The myofiber direction $\mathbf{n}_f$ lies in the $(\mathbf{e}_\eta, \mathbf{e}_\xi)$ plane, and with a fiber angle $\alpha$ measured from $\mathbf{e}_\eta$. The cross-fiber direction $\mathbf{n}_s$ is perpendicular to $\mathbf{n}_f$, and with a sheet angle $\beta$ measured from $\mathbf{e}_h$. The third axis $\mathbf{n}_n = \mathbf{n}_f \times \mathbf{n}_s$ is normal to the plane of myofiber layer.

assumed for $\beta$, that is,

$$\alpha = \alpha_{\text{endo}} t + \alpha_{\text{epi}}(1 - t),$$

$$\beta = \beta_{\text{endo}} t(2t - 1) + 4\beta_{\text{mid}} t(1 - t) + \beta_{\text{epi}}(1 - 2t)(1 - t),$$

$$t = \frac{\lambda - \lambda_{\text{epi}}}{\lambda_{\text{endo}} - \lambda_{\text{epi}}}, \quad \lambda = \sqrt{\left(\frac{x}{a}\right)^2 + \left(\frac{y}{b}\right)^2 + \left(\frac{z}{c}\right)^2}, \tag{14}$$

where $t$ is the relative wall depth measured from the epicardial surface, the angles $(\alpha_{\text{epi}}, \alpha_{\text{endo}}, \beta_{\text{endo}}, \beta_{\text{mid}}, \beta_{\text{epi}})$ are assumed vary linearly from the basal site ($z_{\text{Base}} = 2.5$ cm) to the apical site ($z_{\text{Apex}} = -5.0$ cm), as

$$\begin{aligned}(\alpha_{\text{epi}}, \alpha_{\text{endo}}, \beta_{\text{endo}}, \beta_{\text{mid}}, \beta_{\text{epi}})|_z &= (\alpha_{\text{epi}}, \alpha_{\text{endo}}, \beta_{\text{endo}}, \beta_{\text{mid}}, \beta_{\text{epi}})|_{\text{Apex}} h \\ &\quad + (\alpha_{\text{epi}}, \alpha_{\text{endo}}, \beta_{\text{endo}}, \beta_{\text{mid}}, \beta_{\text{epi}})|_{\text{Base}}(1 - h),\end{aligned} \tag{15}$$

where $h = (z_{\text{Base}} - z)/(z_{\text{Base}} - z_{\text{Apex}})$. Based on the measured distribution of angles $\alpha$ and $\beta$ in canine ventricular myocardium [48], the following architectural parameters are assumed (in degree) for the simulations:

$$\begin{aligned}(\alpha_{\text{epi}}, \alpha_{\text{endo}}, \beta_{\text{endo}}, \beta_{\text{mid}}, \beta_{\text{epi}})|_{\text{Apex}} &= (-40, 80, 10, -18, -20), \\ (\alpha_{\text{epi}}, \alpha_{\text{endo}}, \beta_{\text{endo}}, \beta_{\text{mid}}, \beta_{\text{epi}})|_{\text{Base}} &= (-65, 60, -35, 25, 0).\end{aligned} \tag{16}$$

Figures 3(a) and 3(b) illustrate the transmural distribution of $\alpha$ and $\beta$ at the basal and apical sites, respectively.

### 3.2. *Linear anisotropic viscoelastic passive behavior and active contraction stress*

In the present phantom simulations, a linear anisotropic viscoelastic model is employed for the passive behaviors of normal and ischemic myocardial tissues, and a directional active contraction stress is adopted, that is,

$$\boldsymbol{\sigma} = \frac{\partial W_E}{\partial \boldsymbol{\varepsilon}} + \gamma \mathbf{I} : \dot{\boldsymbol{\varepsilon}} + T_f(\text{Ca}^{2+}, \lambda; T_{\max})\mathbf{n}_f \otimes \mathbf{n}_f, \tag{17}$$

in which the strain energy $W_E$ is calculated on the local structure-based material axes $(\mathbf{n}_f, \mathbf{n}_s, \mathbf{n}_n)$ as

$$\begin{aligned}W_E = \frac{1}{2}\big(&E_{ff}\varepsilon_{ff}^2 + E_{ss}\varepsilon_{ss}^2 + E_{nn}\varepsilon_{nn}^2 + 2E_{fs}\varepsilon_{fs}^2 \\ &+ 2E_{fn}\varepsilon_{fn}^2 + 2E_{ns}\varepsilon_{ns}^2\big),\end{aligned} \tag{18}$$

where $\varepsilon_{ff} = \mathbf{n}_f \cdot \boldsymbol{\varepsilon} \cdot \mathbf{n}_f$, $\varepsilon_{ss} = \mathbf{n}_s \cdot \boldsymbol{\varepsilon} \cdot \mathbf{n}_s$, $\varepsilon_{nn} = \mathbf{n}_n \cdot \boldsymbol{\varepsilon} \cdot \mathbf{n}_n$, $\varepsilon_{fs} = \mathbf{n}_f \cdot \boldsymbol{\varepsilon} \cdot \mathbf{n}_s$, $\varepsilon_{fn} = \mathbf{n}_f \cdot \boldsymbol{\varepsilon} \cdot \mathbf{n}_n$, and $\varepsilon_{ns} = \mathbf{n}_n \cdot \boldsymbol{\varepsilon} \cdot \mathbf{n}_s$. This is a linear approximation to the exponential strain energy employed in the forward heart simulations of Guccione et al. [8] and Usyk et al. [12]. An isotropic Voigt-type viscosity is assumed. The active contraction stress is assumed directly related to the fiber direction $\mathbf{n}_f$. It depends on the current calcium concentration $\text{Ca}^{2+}$ and the relative sarcomere stretch $\lambda$, which is calculated with $\lambda = \mathbf{n}_f \cdot \boldsymbol{\varepsilon} \cdot \mathbf{n}_f + 1$ for small-strain approach. There have been complex models for $T_f$ [49, 50] in heart muscle, and three-dimensional description of active stress $\boldsymbol{\sigma}_f$ [51, 52]. For the purpose of the present DCE phantom simulations, a simplified model of Rachiev and Hayashi [53] is employed:

$$T_f = T_{\max}\frac{\text{Ca}^{2+}}{(\text{Ca}^{2+})_{\max}}\lambda\left(1 - \left(\frac{\lambda - \lambda_0}{\lambda_m - \lambda_0}\right)^2\right), \tag{19}$$

in which $\lambda_0 = 0.8$ and $\lambda_m = 1.2$ are the reference sarcomere stretches for myocardium [54], and $T_{\max}$ is the reference contraction stress.

The material parameters $\mathbf{p} = (E_{ff}, E_{ss}, E_{nn}, E_{ns}, E_{fn}, E_{fs}, \gamma, T_{\max})$ are given for normal and ischemic myocardial

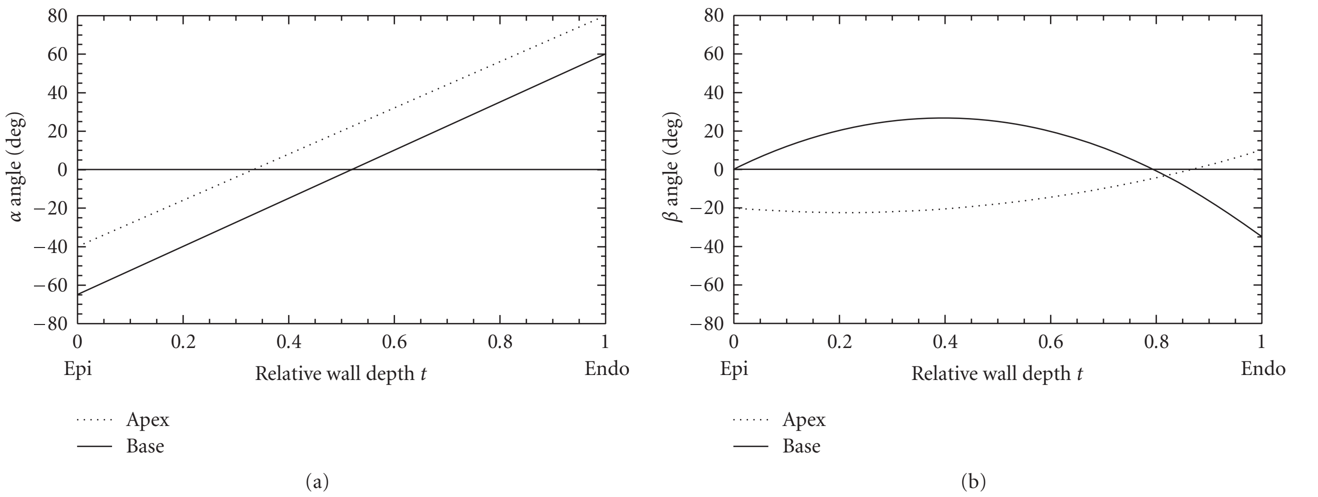

FIGURE 3: Mathematical approximation of the myofiber architecture at basal (solid line) and apical (dotted line) sites of left ventricle model. (a) Linear transmural distribution of fiber angle $\alpha$; (b) quadratic transmural distribution of sheet angle $\beta$.

tissues, respectively, as

$$\mathbf{p}_{\text{Normal}} = (185.0, 35.8, 35.8, 28.0, 28.0, 28.0, 1.0, 800),$$
$$\mathbf{p}_{\text{Ischemic}} = (555.0, 107.4, 107.4, 84.0, 84.0, 84.0, 1.0, 200), \tag{20}$$

where $E$'s and $T_{\max}$ are in kPa, and $\gamma$ is in kPa · second. The ratios between $E$'s reflect the material anisotropy, in consistency with the exponential model of Guccione et al. [8] and Usyk et al. [12]. The magnitudes of $E$'s are chosen such that the end-filling sarcomere stretch lies mostly around 1.1 in the LV wall, and that the ischemic region are three times as stiff as the normal region [4]. For the normal tissue, a reference contractility $T_{\max} = 800$ kPa is chosen for most realistic LV contraction deformation. The ratio between $T_{\max}$ and the passive stiffness $E$'s is consistent with those found in the literature [18]. The contractility of the ischemic myocardium is typically much weaker than the normal tissue [5, 55], and changes with the degree of damage and healing. Therefore, its $T_{\max}$ is assumed at 200 kPa, that is, 25% of the normal tissue. Although the normal tissue is assigned with one set of material parameters, it is heterogeneous owning to the spatial variation of myofiber architecture. So is the ischemic tissue. It is noted that the objective of the present simulations is to investigate the feasibility of cardiac dynamic elastography, instead of the actual mechanical responses in a real heart. Therefore, the phantom, loading conditions, material models, and parameters are not exact.

Material parameters $(E_{ff}, E_{ss}, E_{nn}, E_{ns}, E_{fn}, E_{fs}, \gamma, T_{\max})$ are to be identified in the following DCE reconstructions. The reference stretches $\lambda_0$ and $\lambda_m$ show relatively small variations in different hearts [54], and are assumed known in the simulations. The mass density of myocardium is also assumed known at $\rho_0 = 1g/\text{cm}^3$. Kinetics of $Ca^{2+}$ is described in the next subsection.

### 3.3. LV pressure and calcium kinetics profiles

An empirical LV blood pressure profile $P(t)$ (e.g., [16]) is used, as in Figure 4(a). The passive phase involves rapid/reduced filling (time $t < t_0 = 0.34$ second) and artery contraction ($t_0 \le t < t_1 = 0.48$). The active phase includes LV isovolumetric contraction ($t_1 \le t < t_2 = 0.60$) and rapid/reduced ejection ($t_2 \le t < t_3 = 0.88$). In the active phase, the calcium concentration $Ca^{2+}(t)$ is assumed uniform in the LV wall, and is modeled mathematically as [49]

$$\frac{\text{Ca}^{2+}(t)}{(\text{Ca}^{2+})_{\max}} = \begin{cases} 0 & t \in [0, t_1], \\ \dfrac{t - t_1}{t_2 - t_1} & t \in [t_1, t_2], \\ \dfrac{1 - s}{1 + ks}, \quad s = \dfrac{t - t_2}{t_3 - t_2} & t \in [t_2, t_3], \end{cases} \tag{21}$$

where $k = 10$, as in Figure 4(b). In practice, LV blood pressure and electromechanical activity in an individual heart can be obtained noninvasively with established techniques [56, 57], and are used for DCE reconstructions.

### 3.4. Dynamic deformation of LV

In the phantom simulations, the endocardial surface is subjected to uniform blood pressure $P(t)$ (Figure 4(a)), and the epicardial surface is force free. The basal surface ($z = 2.5$ cm) is fixed, that is, $\mathbf{u} = \mathbf{0}$ at $z = 2.5$ cm. With material model (17)–(20), the dynamic deformation of LV in the time interval $[0, t_3]$ is obtained. The ventricular deformations at $t = 0.4$ second (passive phase) and 0.6 second (active phase) are plotted in Figures 5(a) and 6(a), respectively. Simulations are also

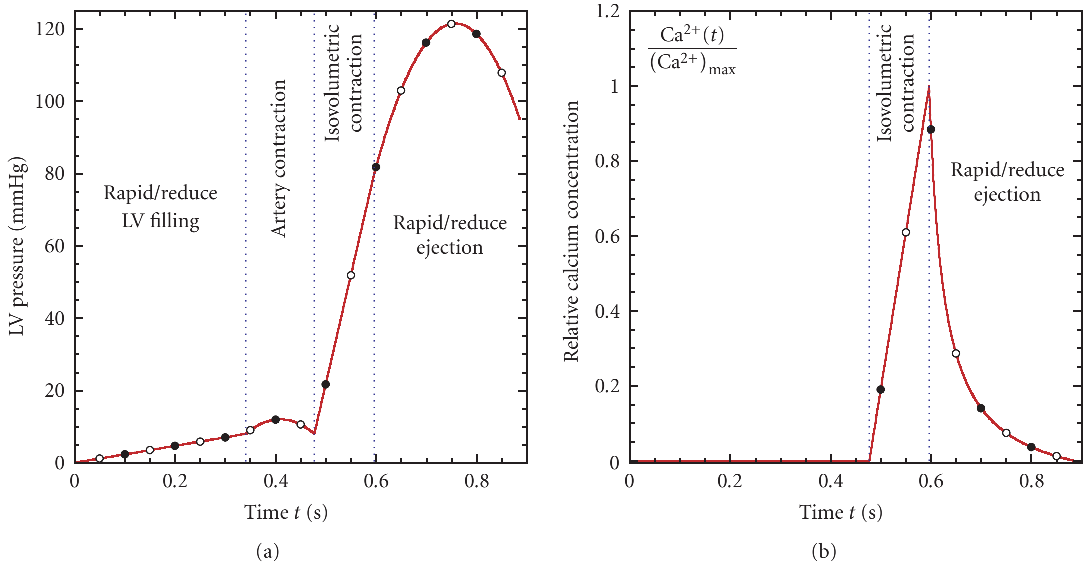

Figure 4: (a) A model blood pressure profile $P(t)$ in the left ventricle, in mmHg. The dots represent time steps when displacement $\mathbf{U}^m$ is measured. Solid dots: time $t = 0.1, 0.2, \ldots, 0.8$ second; open dots: $t = 0.05, 0.15, \ldots, 0.85$ second. (b) A model relative calcium kinetics curve $Ca^{2+}(t)/(Ca^{2+})_{\max}$ in the left ventricular wall.

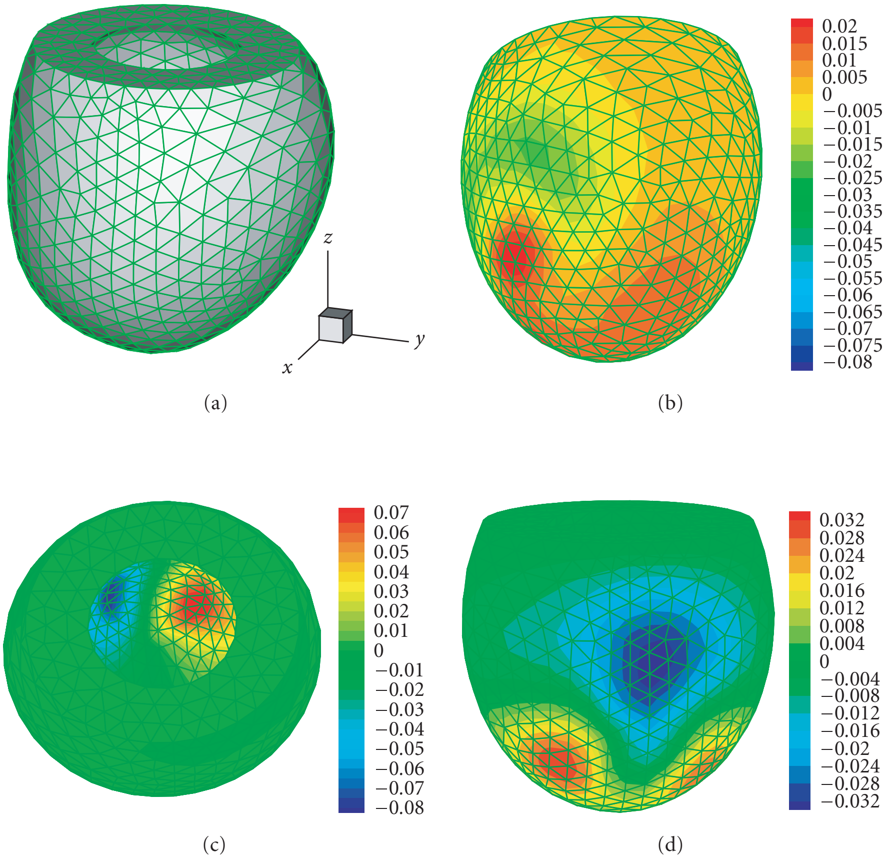

Figure 5: (a) Deformation of ischemic LV phantom at $t = 0.4$ second (passive phase); (b), (c), and (d) differences of epicardial/endocardial displacement components $u_x$, $u_y$, and $u_z$, respectively, between ischemic and normal LV phantoms. View angles and contour levels are chosen for best illustration.

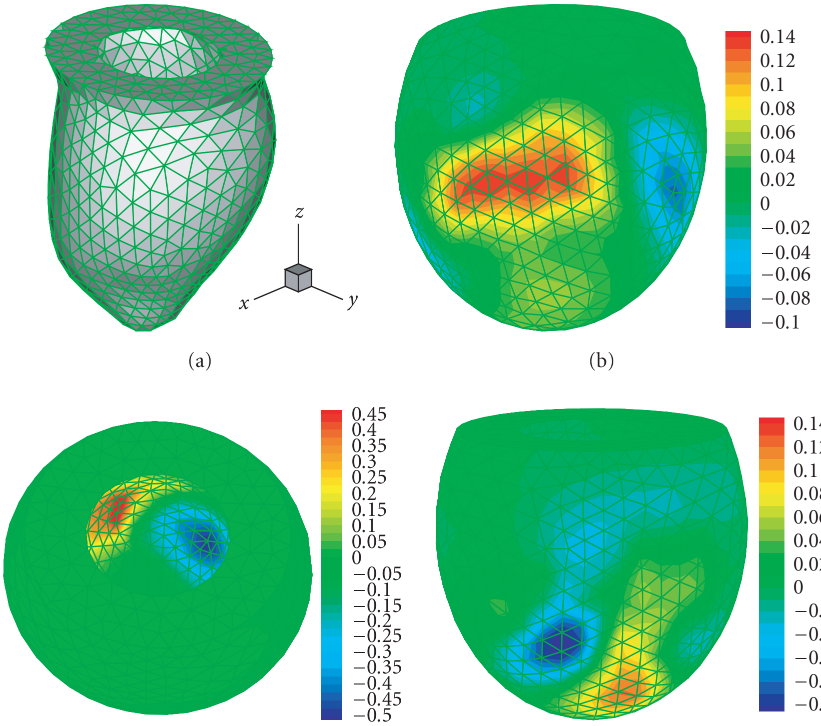

(a) (b) (c) (d)

FIGURE 6: Same as Figure 5, but at $t = 0.6$ second (active phase).

made with a normal phantom without the ischemic region. The differences of epicardial/endocardial displacement components, $u_x$, $u_y$, and $u_z$, between ischemic and normal phantoms are plotted in Figures 5(b), 5(c), and 5(d) for $t = 0.4$ second and Figures 6(b), 6(c), and 6(d) for $t = 0.6$ second, respectively. The comparison shows that the ischemic region, which is passively stiffer and actively weaker, changes the displacement characteristics. In other words, the material parameters of normal and ischemic tissues may be extracted from the displacement measurements.

### 3.5. DCE reconstruction for the passive viscoelastic parameters

The DCE reconstruction is divided into two steps. First, the viscoelastic parameters $E$'s and $\gamma$ are identified using the epicardial/endocardial displacement measured in the passive phase, where no active contraction occurs in the LV wall. Then, the active contraction stress $T_{\max}$ is reconstructed using the displacement in the active phase. The reconstruction follows the optimization procedure (Figure 1), and uses the proposed adjoint gradients (9), (10).

The epicardial/endocardial displacement is measured at time $t = 0.1, 0.2, 0.3, 0.4$ second (Figure 4(a)). The initial guess is picked near to the real parameters of the normal tissue, in order to reach the global minimum of the objective function $\Phi(\mathbf{p})$, which corresponds to the exact material parameters. In practice, the range of the normal tissue parameters may be obtained from available database. Therefore, such an initial guess is feasible. The results are presented in row "Ideal Input" of Table 1. The reconstruction converges after about 250 iteration steps. It yields the exact passive viscoelastic parameters $\mathbf{p}_{\text{Normal}}$ for the normal tissue, and very accurate $\mathbf{p}_{\text{Ischemic}}$ for the ischemic tissue. Very fast convergence of $\mathbf{p}_{\text{Normal}}$ is observed. For instance, parameters $(E_{ff}, E_{ss}, E_{nn}, E_{ns}, E_{fn}, E_{fs}; \gamma)$ of normal tissue reach $(188.8, 37.45, 37.56, 28.14, 26.82, 29.39; 0.844)$ at the 25th iterative step, and $(185.59, 36.24, 36.33, 28.35, 28.06, 28.85; 0.922)$ at the 50th step, then the main adjustment is for the viscosity coefficient $\gamma$. For the ischemic tissue, $E_{ff}$ climbs steadily from the initial guess 125.0, reaches the highest point of 680 at around the 200th step, and decreases gradually to the result of 559.3. The other parameters in $\mathbf{p}_{\text{Ischemic}}$, however, experiences fluctuations. There seems to be a combining effect of the elasticity $E$'s and viscosity $\gamma$ in the monotonous LV filling motion, that is, both contribute to the *effective* stiffness. The weakening effect owning to low elasticity $E$'s may be balanced by a high viscosity $\gamma$. During the reconstruction, $E$'s and $\gamma$ both growth from the initial guess, with a much higher increasing rate for the latter. After about 150 steps, when $\mathbf{p}_{\text{Normal}}$ have converged very closely to the exact values, the ischemic $\gamma$ begins to decrease from the highest value of about 6.6, and the $E$'s increase with faster speed toward the exact values. For the ischemic tissue, the faster convergence

Table 1: Guesses and reconstruction results for dynamic cardiac elastography: passive parameters.

| | Normal myocardium $\mathbf{p}_{\text{Normal}}$ | | | | | | | Ischemic myocardium $\mathbf{p}_{\text{Ischemic}}$ | | | | | | |
|---|---|---|---|---|---|---|---|---|---|---|---|---|---|---|
| | $E_{ff}$ | $E_{ss}$ | $E_{nn}$ | $E_{ns}$ | $E_{fn}$ | $E_{fs}$ | $\gamma$ | $E_{ff}$ | $E_{ss}$ | $E_{nn}$ | $E_{ns}$ | $E_{fn}$ | $E_{fs}$ | $\gamma$ |
| Real | 185.0 | 35.80 | 35.80 | 28.00 | 28.00 | 28.00 | 1.00 | 555.0 | 107.4 | 107.4 | 84.00 | 84.00 | 84.00 | 1.00 |
| Guess | 125.0 | 50.00 | 50.00 | 25.00 | 25.00 | 25.00 | 1.50 | 125.0 | 50.00 | 50.00 | 25.00 | 25.00 | 25.00 | 1.50 |
| DCE reconstruction results | | | | | | | | | | | | | | |
| Ideal input | 185.0 | 35.80 | 35.79 | 28.01 | 28.01 | 28.00 | 1.00 | 559.3 | 101.2 | 105.7 | 83.37 | 82.27 | 81.79 | 1.19 |
| Errors-F | 184.9 | 35.83 | 35.77 | 28.01 | 27.92 | 27.96 | 1.00 | 593.8 | 93.51 | 104.7 | 85.22 | 82.56 | 80.53 | 1.10 |
| Errors-D | 184.9 | 35.69 | 35.88 | 28.08 | 28.12 | 27.87 | 0.99 | 581.1 | 86.14 | 95.6 | 78.08 | 76.50 | 75.72 | 2.51 |
| Errors-FD | 184.9 | 35.51 | 35.76 | 28.03 | 27.97 | 27.76 | 1.07 | 554.5 | 107.5 | 106.2 | 91.49 | 89.22 | 88.68 | 0.43 |
| Static A | 114.7 | 46.40 | 77.19 | 43.91 | 47.05 | 59.74 | — | 326.3 | 613.5 | 130.2 | 0.285 | 33.27 | 19.43 | — |
| Static B | 149.7 | 57.43 | 116.2 | 66.62 | 55.61 | 93.13 | — | 311.9 | 403.5 | 190.4 | 3.988 | 55.99 | 39.72 | — |
| Static C | 131.4 | 52.49 | 99.8 | 57.48 | 50.03 | 79.85 | — | 290.7 | 549.9 | 169.1 | 2.293 | 49.41 | 30.81 | — |

and better accuracy of $E_{ff}$ indicates its relatively stronger role in the LV deformation. The primary deformation pattern in the passive phase is LV dilatation, where the dominant strain-stress relation occurs mainly along the myofiber direction, and the shear strains on the material axes $(\mathbf{n}_f, \mathbf{n}_s, \mathbf{n}_n)$ are relatively weak. Therefore, the influences of $E_{ff}$ are most significant. As a result, $E_{ff}$ for ischemic region is much better identified than the other $E$'s.

There is also size effect, that is, small size of the ischemic region weakens the effects of $\mathbf{p}_{\text{Ischemic}}$ on the overall LV motion. As shown in Figures 5 and 6, deformation variations due to the ischemic region are localized, that is, occur mainly around the ischemic region. This may explain the much faster convergence and higher accuracy for $\mathbf{p}_{\text{Normal}}$ than $\mathbf{p}_{\text{Ischemic}}$. Furthermore, DCE reconstruction has been performed using displacement at nine time steps, that is, $t = 0.05, 0.1, 0.15, \ldots, 0.45$ second (Figure 4(a), both solid and open dots). The improvement is not significant. Better results are expected if transmural displacement field can be measured, for instance, with MRI tagging technique [58]. Reconstructions have also been performed using the direct gradients (11), (12), and similar results are obtained. However, the computational expense is much higher, by a factor of about 5, noting that there are 14 displacement fields $\mathbf{u}_{(i)} = \partial\mathbf{u}/\partial p_i$ to solve (12).

In comparison with our previous static anisotropic elastography simulations [44], the present DCE needs more iterative steps and shows overall lower accuracy. This is due to the approximate numerical scheme for solving the dynamic motion equations (2), (9), that is, Newmark-$\beta$ method in this work.

### 3.6. DCE reconstruction for the active contraction parameters

This section identifies the active contraction stresses $T_{\max}$ (19) for the normal and ischemic tissues, denoted as $T_{\text{Normal}}$ and $T_{\text{Ischemic}}$, respectively. The epicardial/endocardial displacement is measured at time $t = 0.5, 0.6, 0.7, 0.8$ second during the active phase (Figure 4(a)). Two approaches are made.

The first approach employs the exact passive material parameters given in (20). The initial guess is 500.00 for both $T_{\text{Normal}}$ and $T_{\text{Ischemic}}$. It is near to the exact value for the normal tissue. Thirteen iterative steps are needed to get convergent results, $T_{\text{Normal}} = 800.0$ and $T_{\text{Ischemic}} = 200.0$, respectively, which are the exact values. Similar to the above observation on the convergence of $\mathbf{p}_{\text{Normal}}$ and $\mathbf{p}_{\text{Ischemic}}$, $T_{\text{Normal}}$ converges quickly toward the exact value, then $T_{\text{Ischemic}}$ accelerates its convergent speed. More specifically, $T_{\text{Normal}}$ has reached 806.8 at the 8th iterative reconstruction step, while $T_{\text{Ischemic}}$ is at 445.2, about twice of the exact value. Then, $T_{\text{Normal}}$ gradually drops to the exact value, and $T_{\text{Ischemic}}$ approaches rapidly to 200.0. As discussed above for $\mathbf{p}_{\text{Ischemic}}$, two factors probably contribute to the late convergence of $T_{\text{Ischemic}}$, that is, the small size of ischemic region and the low value of $T_{\text{Ischemic}}$ (25% of $T_{\text{Normal}}$).

The second approach uses the previously reconstructed passive viscoelastic material parameters $(E_{ff}, E_{ss}, E_{nn}, E_{ns}, E_{fn}, E_{fs}; \gamma)$ (row "Ideal Input" of Table 1). The pattern of convergence is similar to the above with exact passive parameters, while there are some reconstruction errors. At the 8th iterative step, $T_{\text{Normal}} = 805.6$ and $T_{\text{Ischemic}} = 446.9$. Then $T_{\text{Ischemic}}$ starts to converge quickly. After 13 steps, the gradients $\partial\Phi/\partial\mathbf{p}$ approach zero, and reconstruction results are $T_{\text{Normal}} = 799.5$ and $T_{\text{Ischemic}} = 197.8$.

### 4. DISCUSSIONS

The above DCE reconstructions use exact measurements, that is, exact myofiber architecture, exact epicardial and endocardial displacement, and exact blood pressure. The following simulations investigate the influences of the errors with myofiber architecture and displacement measurements, respectively. Furthermore, a comparison is made between the dynamic cardiac elastography and the previous quasistatic approach.

### 4.1. Errors with myofiber architecture

*In vivo* measurement of the myofiber architecture has been a challenge in cardiac imaging techniques. Recently, Scollan et al. [38] developed a diffusion-tensor MRI to estimate the fiber orientation in a living heart. Another feasible way is to approximate the myofiber architecture using available *ex vivo* experimental database [36, 55, 59–61], based on the understanding that variation of myofiber architecture in individual hearts is relatively small after some anatomy-based mapping.

To simulate the errors with myofiber architecture, randomly picked values between $-5$ and 5 degrees are added to the "exact" fiber angle $\alpha$ and sheet angle $\beta$ (14)–(16) in each element. Reconstruction is then conducted to identify the passive parameters $(E_{ff}, E_{ss}, E_{nn}, E_{ns}, E_{fn}, E_{fs}; \gamma)$. The results are given in the row "Errors-F" of Table 1. It is observed that $\mathbf{p}_{\text{Normal}}$ are not sensitive to the errors. They are $(189.0, 37.57, 37.62, 28.12, 26.75, 29.34; 0.84)$ at the 25th step, and reach $(185.4, 35.68, 36.01, 28.16, 28.02, 28.46; 0.95)$ at the 75th step. Due to the small size and weak influences of the ischemic region on the LV deformation, $\mathbf{p}_{\text{Ischemic}}$ are more sensitive, and show reconstruction errors as large as 10%. The overall patterns of convergence are similar to those of DCE reconstruction with exact myofiber architecture (Section 3.5).

### 4.2. Errors with displacement measurement

Epicardial/endocardial displacement of a beating heart can be computed from dynamic cardiac imaging sequences (Lin & Robb [34], Eusemann et al. [35]), and inevitably contains errors. The response of DCE reconstruction to inaccurate displacement measurement is investigated herein. Randomly generated $-5\%$ to $+5\%$ relative error is added to each "exact" epicardial/endocardial displacement component. The results are shown in the row "Errors-D" of Table 1.

The parameters $\mathbf{p}_{\text{Normal}}$ converge stably, and reach accurate results. In comparison, $\mathbf{p}_{\text{Ischemic}}$ are affected by the displacement errors more significantly. The ischemic parameters $E_{ss}, E_{nn}, E_{ns}, E_{fn}$, and $E_{fs}$ show error as high as about 15%. However, the material anisotropy, described by the ratios between $E$'s is maintained very well, as well as the heterogeneity due to the ratios between $\mathbf{p}_{\text{Normal}}$ and $\mathbf{p}_{\text{Ischemic}}$. The ischemic viscosity parameter $\gamma$ shows the largest error, that is, the result is 2.51, compared to the exact value 1.00. This may probably be explained by the combining effect of $E$'s and $\gamma$, as mentioned above, and the weak influence of ischemic $\gamma$ on the deformation, which may be somehow concealed by the errors with displacement measurements.

DCE reconstruction has also been conducted with both $\pm 5$ degree myofiber architecture errors and $\pm 5\%$ displacement errors. The errors are randomly picked, and are different from the above. The results are given in the row "Errors-FD" of Table 1. In overall, $\mathbf{p}_{\text{Normal}}$ are robust against these errors, with maximum error 7% for $\gamma$. While $\mathbf{p}_{\text{Ischemic}}$ are more sensitive to the measurement errors, the material anisotropy and heterogeneity are well maintained. In fact, the resulting ischemic $E$'s are somehow more accurate than those with displacement errors only, probably due to the differences in the random errors.

Based on our other DCE reconstructions with randomly picked measurement errors, it is shown that the influences of myofiber architecture errors are in general less significant than the displacement errors. This could be explained with concept of *homogenization*, that is, the random myofiber architecture errors at a material point may be compensated by the errors in its neighborhood. In fact, the perturbation on deformation due to material variation at a point is highly localized in a solid. In view of the less accurate reconstruction results for the viscosity parameters, we speculate that methods may be needed to bring out or isolate the viscosity effects, or instance, a modality to use ultrasonic impulse to stimulate a low-magnitude jump-type deformation in the myocardium.

### 4.3. On the quasistatic approach

The previous cardiac elastography studies [28–31] made quasistatic assumption for the LV passive (filling) deformation. Consequently, the effects of inertia and viscosity were omitted. In those studies, the displacement is typically taken near to the highest filling pressure, that is, around $t = 0.4$ or 0.45 second for the present blood pressure profile (Figure 4(a)). To investigate the quasistatic assumption, reconstruction is conducted by deactivating all the terms related to inertia and viscosity in our DCE algorithm and the codes. Exact myofiber architecture data and displacement at $t = 0.4$ and 0.45 second are used. The results are given in the last three rows of Table 1. "Static A" and "Static B" use displacement measured at 0.4 and 0.45 second, respectively, and "Static C" uses displacement at both 0.4 and 0.45 second. Note that the viscosity parameters $\gamma$ cannot be identified with quasistatic assumption.

It is shown clearly that the quasistatic elastography does not yield accurate material parameters for either the normal or the ischemic myocardium. This may indicate the necessity of dynamic description for cardiac elastography. In a real heart, the left ventricle dilates to about the maximum chamber volume near to the end-filling stage, where the velocity field in the wall is overall very low and the effects of viscosity are about minimal. From this point of view, quasistatic elastography is probably a good approach for the elastic parameters $E$'s, and failure of the present quasistatic reconstruction may only indicate that the "real" material parameters we chose do not give almost static deformation at the highest filling blood pressure (around $t = 0.4$ and 0.45 second). On the other hand, it is not clear if the real acceleration field at the end-filling stage is low enough for omitting the inertia effects. Another concern is that, even at the end-filling stage where the overall velocity could be very low, the quasistatic displacement may still be qualitatively

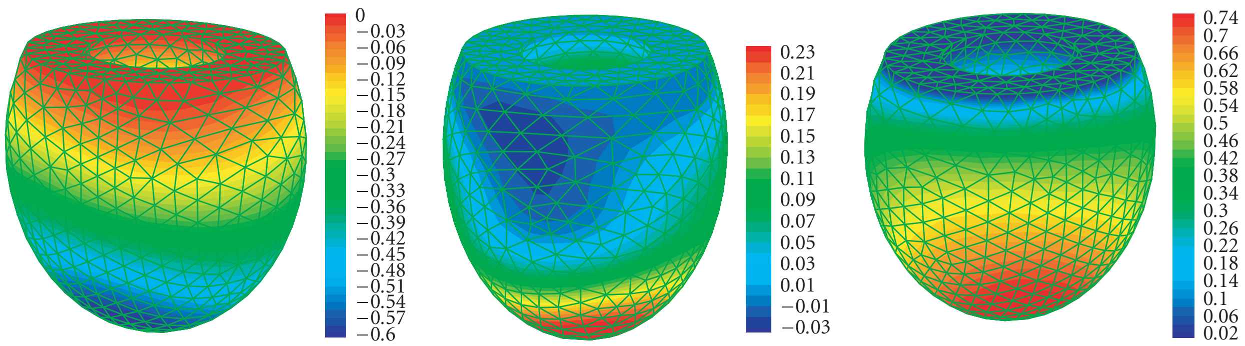

(a) (b) (c)

Figure 7: Differences between dynamic and static displacements on the epicardial surface, at $t = 0.45$ second. (a) Displacement component $u_x$; (b) $u_y$; and (c) $u_z$. View angles and contour levels are chosen for best illustration.

different from the dynamic displacement. Consider a one-dimensional spring-mass-dashpot system, for example, the dynamic maximum displacement (at zero velocity) is higher than the quasistatic maximum displacement under the same force. For the present LV phantom, the differences between dynamic and static displacements on the epicardial surface, with the same material parameters and blood pressure, are given in Figure 7 at $t = 0.45$ second. It is shown that the difference are global and have clear distribution patterns, compared to the much more localized differences due to the ischemic region (Figures 5 and 6), and are much more significant in magnitude than the latter. This suggests that the errors due to quasistatic assumption may overwhelm the effects of the ischemic region. In fact, the quasistatic reconstruction results for $\mathbf{p}_{\text{Ischemic}}$ (Table 1) are qualitatively incorrect. Therefore, quasistatic cardiac elastography needs careful evaluations, particularly using *in vivo* measurements. For the viscosity and active contraction parameters, like $\gamma$ and $T_{\max}$ in the present model, however, the proposed dynamic cardiac elastography is indispensable.

### 5. CONCLUSION

In this work, a dynamic cardiac elastography (DCE) framework has been developed for identification of the material parameters for the passive viscoelastic behaviors and active contractility of myocardium, using time-dependent displacement extracted from dynamic cardiac imaging sequence. The DCE method takes into consideration the myofiber architecture-induced orthotropy and heterogeneity of the material properties. The reconstruction algorithm employs an iterative optimization procedure, and uses user-supplied gradients of the objective function. A dynamic adjoint method is derived for cost-efficient calculation of the gradients. DCE simulations have been conducted to identify the passive and active parameters for normal and ischemic myocardium in a left ventricle phantom. For the normal myocardium, the passive viscoelastic parameters and reference active contraction stress are identified with high accuracy, and are not considerably sensitive to the errors with the myofiber architecture and displacement measurements. For the small ischemic region in the ventricular wall, which is stiffer passively and weaker actively than the normal tissue, the reconstruction results are very accurate with ideal measurements, and are satisfactory when errors are added to the myofiber architecture and displacement measurements. The previous quasistatic assumption for cardiac elastography is investigated. It is suggested that consideration of dynamic deformation may be necessary, particularly for the viscosity and active contraction parameters.

### ACKNOWLEDGMENTS

This work is partially supported by NIH/NIBIB Grant no. EB002667-01 and the University of Iowa Informatics Initiative Grant. The authors would like to thank William Stanford, M.D., University of Iowa, for helpful discussion.

### REFERENCES

[1] American Heart Association, *2004, Heart disease and Stroke Stastistics-2004 update*, Dallas, Texas.

[2] S. Sasayama, H. Nonogi, S. Miyazaki, et al., "Changes in diastolic properties of the regional myocardium during pacing-induced ischemia in human subjects," *Journal of the American College of Cardiology*, vol. 5, no. 3, pp. 599–606, 1985.

[3] M. S. Visner, C. E. Arentzen, D. G. Parrish, et al., "Effects of global ischemia on the diastolic properties of the left ventricle in the conscious dog," *Circulation*, vol. 71, no. 3, pp. 610–619, 1985.

[4] D. D. McPherson, D. J. Skorton, S. Kodiyalam, et al., "Finite element analysis of myocardial diastolic function using three-dimensional echocardiographic reconstructions: application of a new method for study of acute ischemia in dogs," *Circulation Research*, vol. 60, no. 5, pp. 674–682, 1987.

[5] D. K. Bogen, A. Needleman, and T. A. McMahon, "An analysis of myocardial infarction. The effect of regional changes in contractility," *Circulation Research*, vol. 55, no. 6, pp. 805–815, 1984.

[6] R. A. Leyton, "Cardiac ultrastructure and function in the normal and failing heart," in *Cardiac Mechanics: Physiological, Clinical, and Mathematical Considerations*, I. Mirsky, D. N. Ghista, and H. Sandler, Eds., pp. 11–65, John Wiley & Sons, New York, NY, USA, 1974.

[7] L. K. Waldman, Y. C. Fung, and J. W. Covell, "Transmural myocardial deformation in the canine left ventricle: normal in vivo three-dimensional finite strains," *Circulation Research*, vol. 57, no. 1, pp. 152–163, 1985.

[8] J. M. Guccione, K. D. Costa, and A. D. McCulloch, "Finite element stress analysis of left ventricular mechanics in the beating dog heart," *Journal of Biomechanics*, vol. 28, no. 10, pp. 1167–1177, 1995.

[9] M. Nash, *Mechanics and material properties of the heart using an anatomically accurate mathematical model*, Ph.D. thesis, University of Auckland, Auckland, New Zealand, 1998.

[10] J. D. Humphrey, "Continuum biomechanics of soft biological tissues," *Proceedings of the Royal Society A: Mathematical, Physical and Engineering Sciences*, vol. 459, no. 2029, pp. 3–46, 2003.

[11] P. J. Hunter, A. D. McCulloch, and H. E. D. J. ter Keurs, "Modelling the mechanical properties of cardiac muscle," *Progress in Biophysics and Molecular Biology*, vol. 69, no. 2-3, pp. 289–331, 1998.

[12] T. P. Usyk, R. Mazhari, and A. D. McCulloch, "Effect of laminar orthotropic myofiber architecture on regional stress and strain in the canine left ventricle," *Journal of Elasticity*, vol. 61, no. 1–3, pp. 143–164, 2000.

[13] K. D. Costa, J. W. Holmes, and A. D. McCulloch, "Modelling cardiac mechanical properties in three dimensions," *Philosophical Transactions of the Royal Society of London A*, vol. 359, no. 1783, pp. 1233–1250, 2001.

[14] J. C. Criscione, A. D. McCulloch, and W. C. Hunter, "Constitutive framework optimized for myocardium and other high-strain, laminar materials with one fiber family," *Journal of the Mechanics and Physics of Solids*, vol. 50, no. 8, pp. 1681–1702, 2002.

[15] S. J. Sarnoff, E. Braunwald, G. H. Welch Jr., R. B. Case, W. N. Stainsby, and R. Macruz, "Hemodynamic determinants of oxygen consumption of the heart with special reference to the tension-time index," *The American Journal of Physiology*, vol. 192, no. 1, pp. 148–156, 1958.

[16] K.-M. Jan, "Distribution of myocardial stress and its influence on coronary blood flow," *Journal of Biomechanics*, vol. 18, no. 11, pp. 815–820, 1985.

[17] Y. C. Fung, *Biomechanics: Motion, Flow, Stress and Growth*, Springer, New York, NY, USA, 1990.

[18] A. D. McCulloch, L. K. Waldman, J. Rogers, and J. M. Guccione, "Large-scale finite element analysis of the beating heart," *Critical Reviews in Biomedical Engineering*, vol. 20, no. 5-6, pp. 427–449, 1992.

[19] Y. Lanir, "A structural theory for the homogeneous biaxial stress-strain relationships in flat collagenous tissues," *Journal of Biomechanics*, vol. 12, no. 6, pp. 423–436, 1979.

[20] L. L. Demer and F. C. P. Yin, "Passive biaxial mechanical properties of isolated canine myocardium," *The Journal of Physiology*, vol. 339, no. 1, pp. 615–630, 1983.

[21] J. D. Humphrey and F. C. P. Yin, "On constitutive relations and finite deformations of passive cardiac tissue: I. A pseudostrain-energy function," *Journal of Biomechanical Engineering*, vol. 109, no. 4, pp. 298–304, 1987.

[22] A. J. Shacklock, "Biaxial testing of cardiac tissues," M.S. thesis, University of Auckland, Auckland, New Zealand, 1987.

[23] F. C. P. Yin, R. K. Strumpf, P. H. Chew, and S. L. Zeger, "Quantification of the mechanical properties of noncontracting canine myocardium under simultaneous biaxial loading," *Journal of Biomechanics*, vol. 20, no. 6, pp. 577–589, 1987.

[24] P. M. F. Nielsen, P. J. Hunter, and B. H. Smaill, "Biaxial testing of membrane biomaterials: testing equipment and procedures," *Journal of Biomechanical Engineering*, vol. 113, no. 3, pp. 295–300, 1991.

[25] V. P. Novak, F. C. P. Yin, and J. D. Humphrey, "Regional mechanical properties of passive myocardium," *Journal of Biomechanics*, vol. 27, no. 4, pp. 403–412, 1994.

[26] C. A. Phillips and J. S. Petrofsky, "Myocardial material mechanics: characteristic variation of the circumferential and longitudinal systolic moduli in left ventricular dysfunction," *Journal of Biomechanics*, vol. 17, no. 8, pp. 561–568, 1984.

[27] J. H. Omens, D. A. Mackenna, and A. D. McCulloch, "Measurement of strain and analysis of stress in resting rat left ventricular myocardium," *Journal of Biomechanics*, vol. 26, no. 6, pp. 665–676, 1993.

[28] G. J. Han, K. B. Chandran, N. L. Gotteiner, et al., "Application of finite-element analysis with optimisation to assess the in vivo non-linear myocardial material properties using echocardiographic imaging," *Medical and Biological Engineering and Computing*, vol. 31, no. 5, pp. 459–467, 1993.

[29] L. L. Creswell, M. J. Moulton, S. G. Wyers, et al., "An experimental method for evaluating constitutive models of myocardium in in vivo hearts," *American Journal of Physiology, Heart and Circulatory Physiology*, vol. 267, no. 2, part 2, pp. H853–H863, 1994.

[30] M. J. Moulton, L. L. Creswell, R. L. Actis, et al., "An inverse approach to determining myocardial material properties," *Journal of Biomechanics*, vol. 28, no. 8, pp. 935–948, 1995.

[31] N. L. Gotteiner, G. Han, K. B. Chandran, et al., "In vivo assessment of nonlinear myocardial deformation using finite element analysis and three-dimensional echocardiographic reconstruction," *American Journal of Cardiac Imaging*, vol. 9, no. 3, pp. 185–194, 1995.

[32] E. L. Dove, K. P. Phillip, D. D. McPherson, and K. B. Chandran, "Quantitative shape description of left-ventricular cine-CT image," *IEEE Transactions on Biomedical Engineering*, vol. 38, no. 12, pp. 1256–1261, 1991.

[33] J. Duncan, P. Shi, T. Constable, and A. Sinusas, "Physical and geometrical modeling for image-based recovery of left ventricular deformation," *Progress in Biophysics and Molecular Biology*, vol. 69, no. 2-3, pp. 333–351, 1998.

[34] W.-t. Lin and R. A. Robb, "Visualization of cardiac dynamics using physics-based deformable model," in *Medical Imaging 2000: Image Display and Visualization*, vol. 3976 of *Proceedings of SPIE*, pp. 210–217, San Diego, Calif, USA, February 2000.

[35] C. D. Eusemann, E. L. Ritman, M. E. Bellemann, and R. A. Robb, "Parametric display of myocardial function," *Computerized Medical Imaging and Graphics*, vol. 25, no. 6, pp. 483–493, 2001.

[36] I. J. LeGrice, P. J. Hunter, and B. H. Smaill, "Laminar structure of the heart: a mathematical model," *American Journal of Physiology, Heart and Circulatory Physiology*, vol. 272, no. 5, pp. H2466–H2476, 1997.

[37] E. A. Zamir, S. Sheth, J. M. Guccione, K. D. Costa, and L. A. Taber, "A remodeling algorithm for development of transmural cardiac fiber angle distribution," in *Proceedings of ASME Summer Bioengineering Conference*, Big Sky, Mont, USA, June 1999.

[38] D. F. Scollan, A. Holmes, J. Zhang, and R. L. Winslow, “Reconstruction of cardiac ventricular geometry and fiber orientation using magnetic resonance imaging,” *Annals of Biomedical Engineering*, vol. 28, no. 8, pp. 934–944, 2000.

[39] H. Kanai, S.-I. Katsumata, H. Honda, and Y. Koiwa, “Measurement and analysis of vibration in the myocardium telescopic motion for novel echo-graphic diagnosis,” *Acoustical Science and Technology*, vol. 24, no. 1, pp. 17–22, 2003.

[40] J. B. Weaver, E. E. W. Van Houten, M. I. Miga, F. E. Kennedy, and K. D. Paulsen, “Magnetic resonance elastography using 3D gradient echo measurements of steady-state motion,” *Medical Physics*, vol. 28, no. 8, pp. 1620–1628, 2001.

[41] G. I. N. Rozvany, M. P. Bendsøe, and U. Kirsh, “Layout optimization of structures,” *Applied Mechanics Reviews*, vol. 48, pp. 41–119, 1995.

[42] N. Tardieu and A. Constantinescu, “On the determination of elastic coefficients from indentation experiments,” *Inverse Problems*, vol. 16, no. 3, pp. 577–588, 2000.

[43] A. A. Oberai, N. H. Gokhale, and G. R. Feijóo, “Solution of inverse problems in elasticity imaging using the adjoint method,” *Inverse Problems*, vol. 19, no. 2, pp. 297–313, 2003.

[44] Y. Liu, L. Z. Sun, and G. Wang, “Tomography-based 3-D anisotropic elastography using boundary measurements,” *IEEE Transactions on Medical Imaging*, vol. 24, no. 10, pp. 1323–1333, 2005.

[45] A. A. Oberai, N. H. Gokhale, M. M. Doyley, and J. C. Bamber, “Evaluation of the adjoint equation based algorithm for elasticity imaging,” *Physics in Medicine and Biology*, vol. 49, no. 13, pp. 2955–2974, 2004.

[46] D. C. Liu and J. Nocedal, “On the limited memory BFGS method for large scale optimization,” *Mathematical Programming*, vol. 45, no. 1–3, pp. 503–528, 1989.

[47] M. G. Khan, *Encyclopedia of Heart Diseases*, Academic Press, New York, NY, USA, 2005.

[48] K. D. Costa, Y. Takayama, A. D. McCulloch, and J. W. Covell, “Laminar fiber architecture and three-dimensional systolic mechanics in canine ventricular myocardium,” *American Journal of Physiology, Heart and Circulatory Physiology*, vol. 276, no. 2, part 2, pp. H595–H607, 1999.

[49] J. M. Guccione and A. D. McCulloch, “Mechanics of active contraction in cardiac muscle: part I—constitutive relations for fiber stress that describe deactivation,” *Journal of Biomechanical Engineering*, vol. 115, no. 1, pp. 72–81, 1993.

[50] J. M. Guccione, L. K. Waldman, and A. D. McCulloch, “Mechanics of active contraction in cardiac muscle: part II—cylindrical models of the systolic left ventricle,” *Journal of Biomechanical Engineering*, vol. 115, no. 1, pp. 82–90, 1993.

[51] G. I. Zahalak, “Non-axial muscle stress and stiffness,” *Journal of Theoretical Biology*, vol. 182, no. 1, pp. 59–84, 1996.

[52] G. I. Zahalak, V. De Laborderie, and J. M. Guccione, “The effects of cross-fiber deformation on axial fiber stress in myocardium,” *Journal of Biomechanical Engineering*, vol. 121, no. 4, pp. 376–385, 1999.

[53] A. Rachev and K. Hayashi, “Theoretical study of the effects of vascular smooth muscle contraction on strain and stress distributions in arteries,” *Annals of Biomedical Engineering*, vol. 27, no. 4, pp. 459–468, 1999.

[54] I. Mirsky, D. N. Ghista, and H. Sandler, *Cardiac Mechanics: Physiological, Clinical, and Mathematical Considerations*, John Wiley & Sons, New York, NY, USA, 1974.

[55] R. Mazhari and A. D. McCulloch, “Integrative models for understanding the structural basis of regional mechanical dysfunction in ischemic myocardium,” *Annals of Biomedical Engineering*, vol. 28, no. 8, pp. 979–990, 2000.

[56] J. M. Tyszka, D. H. Laidlaw, J. W. Asa, and J. M. Silverman, “Three-dimensional, time-resolved (4D) relative pressure mapping using magnetic resonance imaging,” *Journal of Magnetic Resonance Imaging*, vol. 12, no. 2, pp. 321–329, 2000.

[57] O. P. Faris, F. J. Evans, D. B. Ennis, et al., “A novel technique for cardiac electromechanical mapping with magnetic resonance imaging tagging and an epicardial electrode sock,” *Annals of Biomedical Engineering*, vol. 31, no. 4, pp. 430–440, 2003.

[58] D. B. Ennis, F. H. Epstein, P. Kellman, L. Fananapazir, E. R. McVeigh, and A. E. Arai, “Assessment of regional systolic and diastolic dysfunction in familial hypertrophic cardiomyopathy using MR tagging,” *Magnetic Resonance in Medicine*, vol. 50, no. 3, pp. 638–642, 2003.

[59] D. D. Streeter Jr. and W. T. Hanna, “Engineering mechanics for successive states in canine left ventricular myocardium. I. Cavity and wall geometry,” *Circulation Research*, vol. 33, no. 6, pp. 639–655, 1973.

[60] D. D. Streeter Jr. and W. T. Hanna, “Engineering mechanics for successive states in canine left ventricular myocardium. II. Fiber angles and sarcomere length,” *Circulation Research*, vol. 33, no. 6, pp. 656–664, 1973.

[61] P. M. F. Nielsen, I. J. Le Grice, B. H. Smaill, and P. J. Hunter, “Mathematical model of geometry and fibrous structure of the heart,” *American Journal of Physiology, Heart and Circulatory Physiology*, vol. 260, no. 4, part 2, pp. H1365–H1378, 1991.

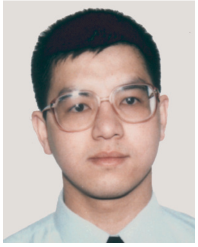

**Yi Liu** is a Research Scientist in Department of Biomedical Engineering at Indiana University Purdue University Indianapolis (IUPUI), Indianapolis. He received his Ph.D. degree of mechanical engineering from University of Pennsylvania (UPENN), in 2003. During 2003–2005, he was a Research Scientist in the Center for *X*-Ray and Optical Tomography in Radiology Department at the University of Iowa (UI). His research interests include micromechanics method for the nonlinear mechanical properties of composites, polycrystals and biological tissues, biomedical imaging and elastography for breast cancer, and cardiovascular abnormality.

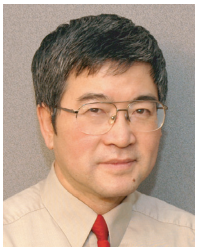

**Ge Wang** is a Professor in Radiology Department at the University of Iowa (UI). In addition, he is the Director of UI’s Center for *X*-Ray and Optical Tomography. He received his Ph.D. degree in electrical and computer engineering from State University of New York, Buffalo, in 1992. He was an Instructor and Assistant Professor with Mallinckrodt Institute of Radiology, Washington University, St. Louis, MO, from 1992–1996. He was an Associate Professor with University of Iowa during 1997–2002. His research interests include computed tomography, bioluminescence tomography, and systems biomedicine. He has authored or coauthored over 300 journal articles and conference papers. He is the Editor-in-Chief for International Journal of Biomedical Imaging, and Associate Editor for IEEE Transactions Medical Imaging and Medical Physics. He is an IEEE Fellow and an AIMBE Fellow. He is also recognized by a number of awards for academic achievements.

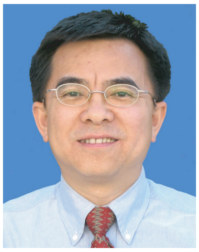

**L. Z. Sun** is an Associate Professor and Vice Chair in Department of Civil and Environmental Engineering at University of California (UCI), Irvine. He has secondary appointment of Associate Professor in UCI, Department of Chemical Engineering and Materials Science. He received his Ph.D. degree in structural mechanics from University of California (UCLA), Los Angeles, in 1998. His main area of research interest is the micro/nanomechanics of heterogeneous composite materials, with applications for civil, mechanical, aerospace, electronic, and biomedical engineering. He has published more than 50 peer-reviewed journal papers in the fields of mechanics and materials, applied physics, and biomedical engineering. He is a Member of ASCE, ASME, MRS, and AAAS.

